# Activation of Nrf2/Keap1 pathway by oral Dimethylfumarate administration alleviates oxidative stress and age-associated infertility might be delayed in the mouse ovary

**DOI:** 10.1186/s12958-019-0466-y

**Published:** 2019-02-13

**Authors:** Nana Akino, Osamu Wada-Hiraike, Wataru Isono, Hiromi Terao, Harunori Honjo, Yuichiro Miyamoto, Michihiro Tanikawa, Kenbun Sone, Mana Hirano, Miyuki Harada, Tetsuya Hirata, Yasushi Hirota, Kaori Koga, Katsutoshi Oda, Tomoyuki Fujii, Yutaka Osuga

**Affiliations:** 1 Department of Obstetrics and Gynecology, Graduate school of Medicine, The University of Tokyo, Tokyo, 1138655 Japan; 2 Department of Obstetrics and Gynecology, Mizonokuchi Hospital, Teikyo University, Kawasaki, 2138507 Japan

**Keywords:** Nrf2/Keap1, Dimethylfumarate, Oxidative stress, Ovarian reserve, Infertility

## Abstract

**Background:**

Age-associated infertility is a problem worldwide, and management of oxidative stress is known to be essential. Nuclear factor-E2-related factor 2 (Nrf2)/Kelch-like ECH-associated protein 1 (Keap1)-antioxidant response element (ARE) signaling pathway works as an essential defense mechanism against oxidative stress, and an oral drug Dimethylfumarate (DMF) is known to activate the pathway.

**Methods:**

We tested the hypothesis that oral DMF could alleviate oxidative stress in the ovary, resulting in salvation of age-associated infertility in a mouse model of reproductive age, and we examined the effects of DMF administration. 20 mg/kg DMF was administrated to female mice from 32 to 48 weeks, and Nrf2 levels, antioxidant levels, ovarian reserve, DNA damage, and oxidative stress were examined.

**Results:**

DMF administration resulted in elevated mRNA and protein levels of Nrf2, antioxidants, and telomere, and serum levels of Nrf2 and anti-mullerian hormone were also elevated. Results of TUNEL assay and Immunohistochemistry of mice ovarian tissues showed that DNA damage and oxidative stress were decreased by DMF administration, and significantly more oocytes were collected along with preservation of 60% more primordial follicles.

**Conclusions:**

Our data suggest that DMF administration activates the Nrf2/Keap1 pathway, elevate levels of antioxidants, and decrease DNA damage and oxidative stress, resulting in improved ovarian reserve in the mouse ovary.

## Background

With the change of lifestyles in developed countries, more women tend to delay childbearing until their late 30s and 40s [1, 2]. In many developed countries, a women’s average age at first childbirth is approximately 28–30 years, and percentage of maternal age over 35 years old increased from 7.9 to 20.2% in UK (1986 → 2008), 4.7 to 14% in USA (1980 → 2005), and 8.5 to 24.8% in Australia (1987 → 2008) [3]. Advanced age plays a fundamental role in infertility, and the percentage of couples receiving infertility treatment is increasing by the year [1, 4]. However, despite public misconceptions, age-associated decline in fertility is difficult to overcome even using IVF techniques, and only half of couples with female age at 30–35, one third of that with 35–40, and only 10% over age of 40 conceive using IVF treatment [5–8]. Therefore, treatment to delay age-associated infertility would be an innovative solution to avoid heavy-burden IVF treatment for considerable couples.

The relationship between age-associated infertility and oxidative stress (OS) is now well established [9]. OS is a state characterized by an imbalance between reactive oxygen species (ROS) and antioxidant scavenger enzymes [10]. In a healthy body the production of ROS and antioxidants are balanced, but when imbalance occurs, the cells are threatened by damaging of lipids, proteins, and nucleic acids [11]. Though adequate amount of ROS is known to be necessary for normal ovulation [12], exposure of excess ROS is unfavorable for oocytes, resulting in infertility. From these facts, management of OS may be effective in protecting the ovary from aging, and an effective approach to overcome infertility.

We previously focused on activation of Nuclear factor-E2-related factor 2 (Nrf2)/Kelch-like ECH-associated protein 1 (Keap1)-antioxidant response element (ARE) signaling pathway and reduction of OS in human granulosa cells [13]. Nrf2/Keap1 pathway is one of the most important mechanisms against OS [14–16], and during normoxia, Nrf2, a transcription factor with high sensitivity to OS, is held in the cytoplasm and maintained at low levels by an inhibitory protein; Keap1 [17]. Once OS occurs, Nrf2 dissociates from Keap1 and translocated into the nucleus, which results in its binding to specific DNA sequence ARE and transcription of downstream target genes [14], and antioxidants including catalase and superoxide dismutase (SOD) are elevated [18]. Enhanced Nrf2 signaling is associated with protection against a wide array of diseases such as cancer [19], diabetes [20], and neurodegenerative diseases [21].

Dimethylfumarate (DMF) is an activator of the Nrf2 pathway, and our previous study showed that DMF alleviated OS in human granulosa cells [22]. Although the exact mechanism remains unclear [23], DMF is recently used as a new first-line oral drug to treat relapsing forms of multiple sclerosis (MS) in USA, Europe, and Japan [14, 24, 25], and studies show that DMF reduces ROS production, and antioxidants were elevated in neurons [26]. These facts are leading to investigation on relation of DMF and OS and inflammatory related diseases, such as Alzheimer’s disease, Parkinson’s disease, chronic pulmonary disease, asthma, diabetes, and rheumatoid arthritis [27–31].

Therefore, we hypothesized that DMF administration could alleviate OS in the ovary, resulting in salvation of age-associated infertility in a mouse model of reproductive age, and the effects of DMF administration were examined.

## Materials and methods

### Animals

Experimental procedures for mice were approved by the animal experiment committee of The University of Tokyo (authorization reference number: P17–009). 20-week-old virgin female BALB/c mice (weighing 25-30 g) were purchased from Charles River Laboratories, Inc. (Kanagawa, Japan) and were bred to 32 weeks prior to experimental procedures and sacrificed at 48 weeks. The mice were housed in a temperature-controlled room with 12-h dark/like cycles, with free access to pelleted food (CLEA rodent diet CE-2, Japan CLEA, Tokyo, Japan) and water.

### Study design

**Fig. 1:**
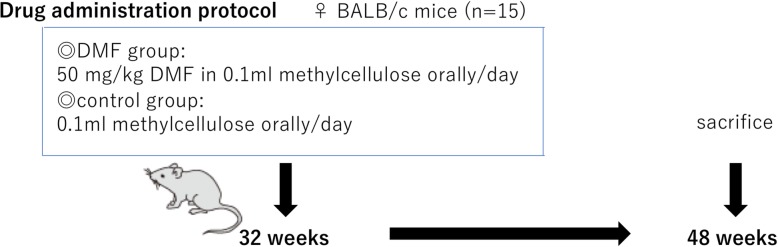
Drug administration protocol. DMF group mice received 20 mg/kg DMF dissolved in 0.1 ml methylcellulose orally/day, and control group mice received 0.1 ml methylcellulose orally/day, from 32 to 48 weeks until the day before sacrifice

In total, 30 mice were randomly allocated into two groups with *n* = 15 in each group. The dose of DMF (Sigma Aldrich, Darmstadt, Germany) was selected carefully from available literatures [24, 32–34], and animal studies concerning toxicity and adverse events on DMF administration (https://tec.ms-supportnavi.com/content/dam/commercial-jp/neurology/mssupportnavi/tecfidera/pdf/product/tecfidera_interview.pdf). As shown in Fig.1, DMF-treated group mice received 50 mg/kg DMF dissolved in 0.1 ml methylcellulose orally once a day for 16 weeks (from 32 weeks to 48 weeks) until the day before sacrifice, and control group mice received 0.1 ml methylcellulose orally once a day for the same period.

### Oocyte and tissue collection

To count oocytes, cumulus-oocyte complexes (COC) were collected from female mice via controlled ovarian stimulation (COS) 13 to 18 h after the injection of human chorionic gonadotropin (hCG; ASKA Pharmaceutical Co, Ltd., Tokyo, Japan). In all mice, COS was performed by intraperitoneal (IP) injection of 7.5 IU pregnant mare’s serum gonadotropin (ASKA Animal Health Co, Ltd., Tokyo, Japan) followed by IP injection of 7.5 IU hCG 47 to 49 h later. The ampulla of the fallopian tube was observed to confirm ovulation. Under anesthesia, which was achieved with pentobarbital sodium at a dosage of 50 mg/kg body weight (Somnopentyl; Kyoritsu Seiyaku Corporation, Tokyo, Japan), both oviducts and ovaries were surgically removed, and the COCs were collected into 60 μL of human tubal fluid (HTF, MR-070-D, EmbryoMax HTF × 1; Merck Millipore, Darmstadt, Germany). Before female mice were sacrificed, a 3- to 6-month-old BALB/c male mouse was sacrificed and sperm were expelled from the cauda epididymis of the male mouse into 200 μL of HTF and incubated for at least 30 min to denature granulosa cells consisting the cumulus by hyaluronidase secreted by sperm at fertilization, for accurate follicle count. Approximately 2 μL of the sperm suspension at a concentration of 6 to 7 × 10^5^ sperm/mL was used to inseminate the oocytes in the aforementioned 60 μL drop of HTF, and the number of oocytes collected from each mouse was confirmed visually under an electronic microscope.

### Histology and follicle count

Mouse ovaries were fixed in 10% neutral buffered formalin, embedded in paraffin, and sectioned at a thickness of 20 μm. Sections were stained with hematoxylin and eosin (H&E-stain). We slightly modified the method introduced [35, 36] to count the numbers of follicles in the ovaries. In brief, Ovarian follicles were classified as primordial (oocytes with single layer of flattened granulosa cells), primary (oocytes with single layer of cuboidal or mixed cuboidal/flattened granulosa cells), secondary (oocytes with more than one layer of granulosa cells) or antral (oocytes with multiple layers of granulosa cells and possessing an antral space or spaces) and were further classified as healthy or atretic. Primordial and primary follicles were counted in every serial section, secondary follicles in every 3rd serial section, and antral follicles in every 5th serial section, taking care to only to count each of these structures once.

### Elisa

Blood samples were collected via cardiac puncture when mice were sacrificed, and a serum separator tube (BD Microtainer, NJ, USA) was used to allow samples to clot overnight at 4 °C before centrifugation for 15 min at 1000×g. Serum concentration of anti-mullerian hormone (AMH) and Nrf2 was measured using the Mouse AMH enzyme-linked immunosorbent assay (ELISA) kit (CSB-E13156m, CUSABIO Life science, Wuhan, China) for AMH, and Mouse Nrf2 ELISA kit (CSB-E16188m, CUSABIO Life Science) for Nrf2, according to the manufacture’s procedures. For both procedures, mouse serum was diluted 10-fold with distilled water and subjected to assay. The absorbance was measured at 450 nm using an Epoch multivolume spectrophotometer system (BioTek, Vermont, USA). All samples were analyzed in triplicate.

### RNA extraction and real-time qPCR

Total RNA was extracted from ovaries of mice using ISOGEN (NIPPON GENE, Tokyo, Japan). One microgram of total RNA was reverse transcribed using the ReverTra Ace quantitative polymerase chain reaction (qPCR) RT Master Mix with genomic DNA remover (TOYOBO, Osaka, Japan) in a volume of 40 μL. For the quantification of various mRNA levels, quantitative real-time PCR was performed using the Light Cycler System (Roche Molecular Biochemicals, Mannheim, Germany). The genes, Nrf2, Catalase, SOD1, NAD(P)H quinone dehydrogenase 1 (NQO1), Telomere, and telomerase reverse transcriptase (TERT) were examined, and glyceraldehyde-3-phosphate dehydrogenase (GAPDH) mRNA was used as an internal standard for RNA loading. The primer sequences are as follows: Nrf2 (gene accession number: NM_031789.2; sense, 5’-GGTTGCCCACATTCCCAAAC-3′; antisense, 5’-TCCTGCCAAACTTGCTCCAT-3′), Catalase (gene accession number: NM_012520.1; sense, 5’-TTGACAGAGAGCGGATTCCT -3′; antisense, 5’-AGCTGAGCCTGACTCTCCAG-3′), SOD1 (gene accession number: NM_017050.1; sense, 5′- CGGATGAAGAGAGGCATGTT-3′; antisense, 5’-CACCTTTGCCCAAGTCATCT-3′), NQO1 (gene accession number: NM_022503.1; sense, 5’-GCAGGATTTGCCTACACAATATGC-3′; antisense, 5′- AGTGGTGATAGAAAGCAAGGTCTTC-3′), Telomere (gene accession number: NT_039202.7; sense, 5′- CGGTTTGTTTGGGTTTGGGTTTGGGTTTGGGTTTGGGTT-3′; antisense, 5′- GGCTTGCCTTACCCTTACCCTTACCCTTACCCTTACCCT-3′), TERT (gene accession number: NM_009354.1; sense, 5′- GGATTGCCACTGGCTCCG-3′; antisense, 5′- TGCCTGACCTCCTCTTGTGAC-3′), and GAPDH (gene accession number: NM_017008; sense, 5’-TCCACCACCCTGTTGCTGTA-3′; antisense, 5′- ACCACAGTCCATGCCATCAC-3′). The PCR conditions were as follows: 40 cycles at 98 °C for 10 s, 60 °C for 10 s, and 68 °C for 30 s. 15 individual mice were used in one independent experiment, and all samples were analyzed in triplicate.

### Western blotting

Mouse ovaries were minced and lysed in lysis buffer (Cell Signaling Technology, Massachusetts, USA) containing phosphatase inhibitors (Nacalai Tesque, Kyoto, Japan) and protease inhibitors (Roche), and insoluble material was removed by centrifugation at 14000 m/sec, for 12 min at 4 °C. The supernatants were recovered, and the protein concentrations were measured using Bio-Rad protein assay reagent (Bio-Rad Laboratories, Hercules, CA). Equivalent amounts of lysate protein (10 μg) were subjected to Mini-PROTEAN TGX Precast Protein Gels (Bio-Rad) and electrophoretically transferred onto Trans-Blot Turbo Transfer Packs (Bio-Rad) using Trans-Blot Turbo Transfer System (Bio-Rad).

After blocking with 10% fat-free powdered milk in phosphate buffered saline (PBS) at room temperature for 1 h, the membranes were blotted overnight at 4 °C with primary antibodies, including anti-Nrf2 (1:100; 16,396–1-AP, Proteintech Group, Illinois, USA), anti-Keap1 (1:1000; ab66620, Abcam Ltd., Cambridge, UK), anti-catalase (1:200; ab16731, Abcam Ltd.), anti- SOD1 (1:1000; ab13499, Abcam Ltd.), anti- NQO1 (1:1000; SC-32793, Santa Cruz, Texas, USA), and anti-TERT (1:100; LS-B9932, LSBio, Washington, USA). Then, the blots were incubated with the appropriate secondary antibodies (anti-rabbit Immunoglobulin G (IgG), 7074S, 1:3000; anti-mouse IgG, 7076S; 1:3000, Cell Signaling) at room temperature for 1 h and developed using ECL Plus Western blotting (WB) detection reagents (GE Healthcare, Buckinghamshire, UK). The images were scanned by the luminescent image analyzer Image Quant LAS 4000 mini (GE Healthcare). The expression of target proteins was internally normalized to the optical density of β-actin (1:2000; A2228, Sigma Aldrich) by the Image J software (http://rsb.info.nih.gov/ij/). All samples were analyzed in triplicate, and representative blots are shown in Figs. 5 and 8.

### Immunohistochemistry

Mouse ovary tissue sections were immunostained with an anti-Nrf2 antibody (1:200, 16,396–1-AP, Proteintech Group), and anti- Human 8-hydroxy-2′-desoxyguanosine (8-OHdG) antibody (1:100, N45.1, JaICA, Shizuoka, Japan) using the EnVision + Dual Link System/HRP (3,3-diaminobenzidine (DAB)) Kit (Dako, Tokyo, Japan). Isotype-specifc IgG served as the negative control. Antigen retrieval was performed using target retrieval solution (Dako).

### Terminal deoxynucleotidyl transferase (TdT) dUTP Nick-end labeling (TUNEL) assay

An in situ cell death detection peroxidase (POD) Kit (Cat. No. 11684817910, Roche) was used for the TUNEL (Terminal deoxynucleotidyl transferase (TdT) dUTP Nick-End Labeling) technique, and mouse ovary tissue sections were stained according to the manufacturer’s protocol for paraffin-embedded tissues. Briefly, the sections were deparaffinized in xylene and rehydrated in alcohol. They were then incubated with 20 μg/ml proteinase K solution (Roche) for 8 min at room temperature. The tissues were then deparaffinized with PBS two times for 3 min. After washing with PBS, the tissues were immersed in 3% hydrogen peroxide in methanol for 15 min to block endogenous peroxidase activity. The sections were deparaffinized with PBS and incubated with TUNEL solution (450 μL label solution, 50 μL of enzyme solution) (Cat. No. 11684817910, Roche) for 60 min at room temperature in a moist and dark environment. The remaining 100 μL of label solution was used for negative controls for alternate sections. The tissues were then deparaffinized with PBS three times for 3 min. Subsequently, the tissues were incubated with converter-POD solution in a humidified environment at room temperature for 30 min. Tissues were then deparaffinized again with PBS three times for 3 min. After washing, TUNEL positive cells were stained with DAB as the chromogen, and the slides were counterstained with Mayer’s hematoxylin. Stained slides were subjected to a decreasing alcohol series. Finally, after incubation for 15 min in xylene, slides were mounted.

### Statistical analysis

Statistical analyses were performed using the JMP Pro 11 software (SAS Institute, North Carolina, USA). All results are shown as mean ± standard error of the mean. Data were analyzed using Student *t* test for paired comparison. A *p* value < 0.05 was considered statistically significant.

## Results

### DMF administration resulted in increased oocyte collection and preservation of primordial follicles

No adverse events were observed throughout the whole period of drug administration to both groups, and the weight of mice at 12 months showed no significant difference between the DMF and control group (DMF group: 28.9 ± 0.43 g vs control group: 29.1 ± 0.43 g, *p* = 0.76).

**Fig. 2:**
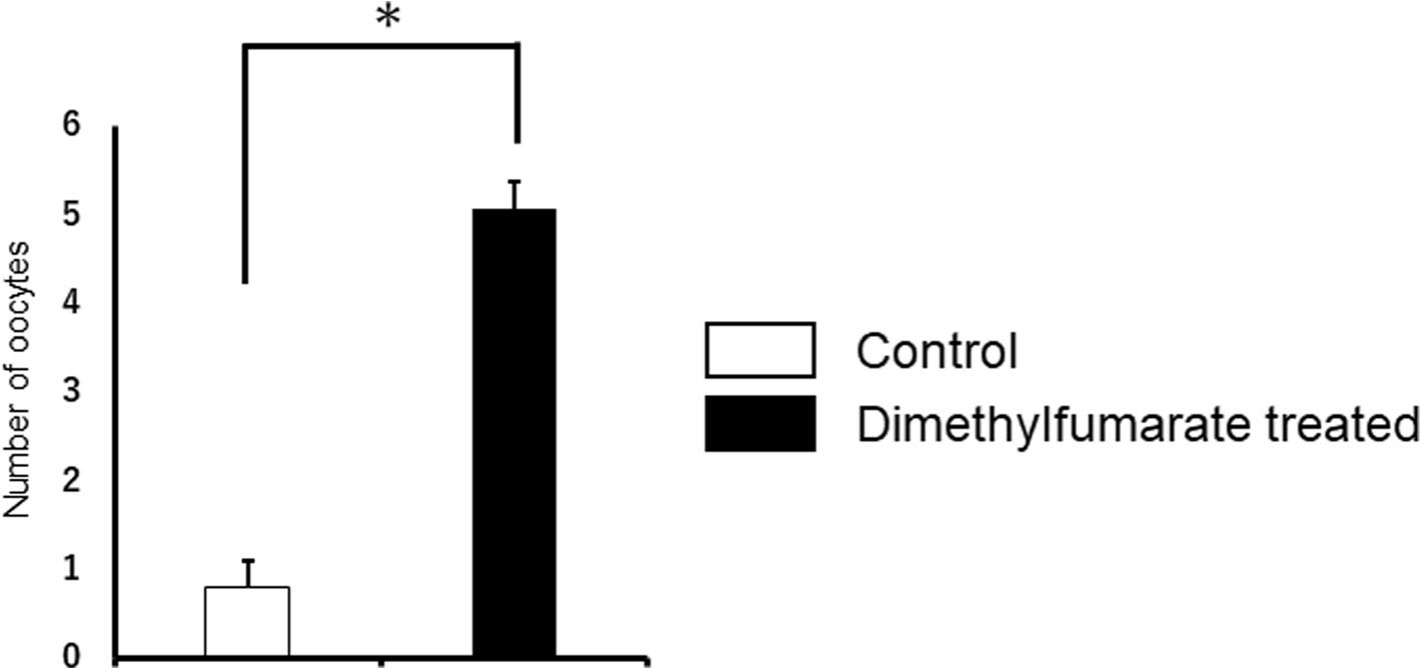
Number of oocytes collected after COS. DMF administration resulted in significant difference in number of oocyte collection (5.1 ± 0.3 oocytes per mouse), compared to the control group (0.8 ± 0.3 oocyte per mouse), indicating the positive effect of DMF on ovarian reserve. *p* < 0.001. The number of samples was *n* = 15 each

Our previous studies show that number of oocytes collected after COS show an age-associated decline, and approximately only 20% of oocytes are collected at 12 months age (5.9 ± 0.6), compared to 2–3 month age (25.2 ± 2.3) [37]. As shown in Fig. 2, DMF administration resulted in significant difference in number of oocyte collection (5.1 ± 0.3 oocytes per mouse), compared to the control group (0.8 ± 0.3 oocyte per mouse), indicating the positive effect of DMF on ovarian reserve.

**Fig. 3:**
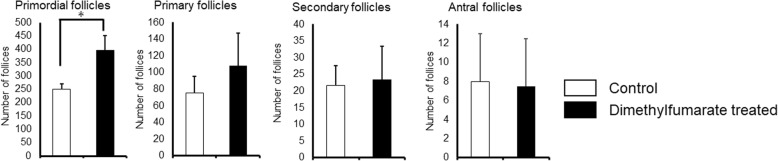
Number of follicles per ovary (H&E-stain). Number of follicles of various stages of development per ovary is shown. The number of follicles showed no significant difference in primary follicles (DMF group: 108.2 ± 38.7 vs control group: 75.2 ± 14.3, *p* = 0.12), secondary follicles (DMF group: 23.5 ± 10.0 vs control group: 21.6 ± 5.9, *p* = 0.72), and antral follicles (DMF group: 7.5 ± 3.7 vs control group: 8.0 ± 1.5, *p* = 0.81), but approximately 60% more primordial follicles (DMF group: 395.0 ± 15.8 vs control group: 251.2 ± 17.3, *p* = 0.0002) were preserved in DMF group. The number of samples was *n* = 6 each

Age-associated ovarian follicle decline was also evident from H&E-stained ovarian tissue sections previously [37], and number of primordial follicles, primary follicles, secondary follicles, and antral follicles per ovary were confirmed in DMF and control groups (Fig. 3). The number of follicles showed no significant difference in primary follicles (DMF group: 108.2 ± 38.7 vs control group: 75.2 ± 14.3, *p* = 0.12), secondary follicles (DMF group: 23.5 ± 10.0 vs control group: 21.6 ± 5.9, *p* = 0.72), and antral follicles (DMF group: 7.5 ± 3.7 vs control group: 8.0 ± 1.5, *p* = 0.81), but approximately 60% more primordial follicles (DMF group: 395.0 ± 15.8 vs control group: 251.2 ± 17.3, *p* = 0.0002) were preserved in DMF group.

### DMF elevated serum AMH and Nrf2 levels

**Fig. 4:**
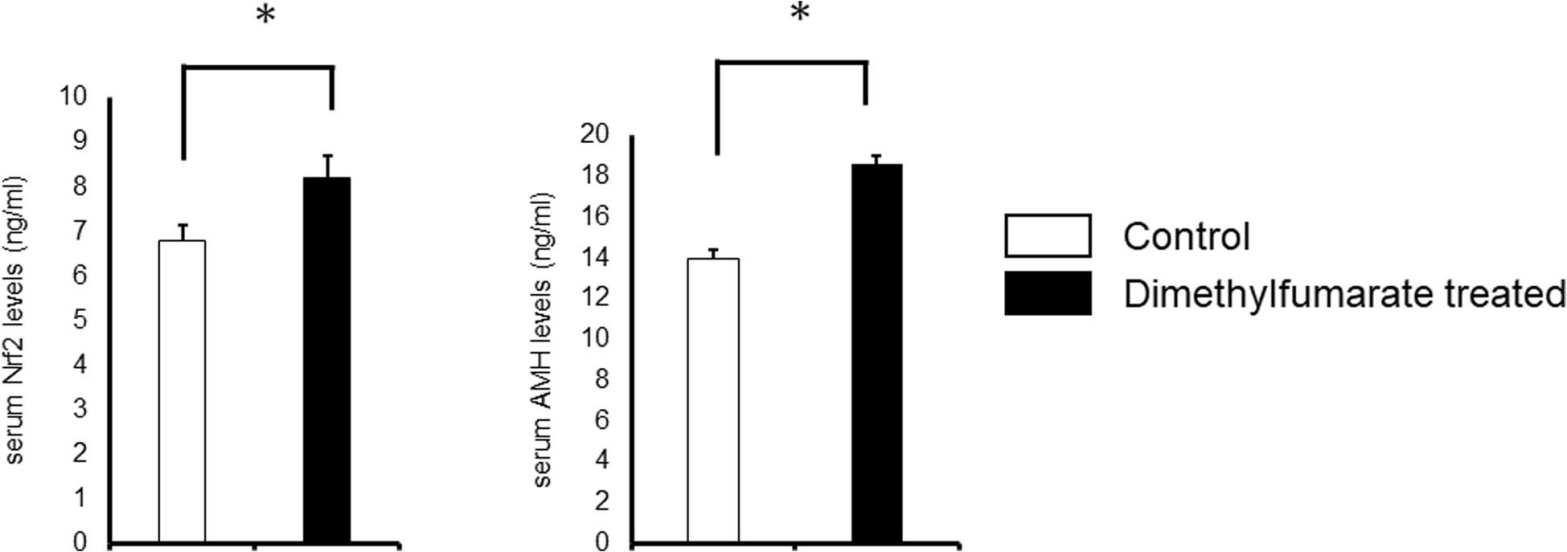
Serum Nrf2 and AMH levels. Serum Nrf2 (control group: 6.79 ± 0.12 ng/ml vs DMF group: 8.21 ± 0.11 ng/ml, *p* < 0.0001) and AMH (control group: 13.93 ± 0.44 ng/ml vs DMF group: 18.63 ± 0.38 ng/ml, *p* < 0.0001) levels were significantly higher in DMF group compared to control group. The number of samples was n = 15 each

To confirm the effect of DMF administration, the levels of AMH and Nrf2 of serum were measured. As shown in Fig. 4, both AMH (control group: 13.93 ± 0.44 ng/ml vs DMF group: 18.63 ± 0.38 ng/ml, *p* < 0.0001) and Nrf2 (control group: 6.79 ± 0.12 ng/ml vs DMF group: 8.21 ± 0.11 ng/ml, p < 0.0001) levels were significantly higher in DMF group compared to control group.

### mRNA and protein levels of Nrf2 and anti-oxidants were elevated by DMF administration

**Fig. 5:**
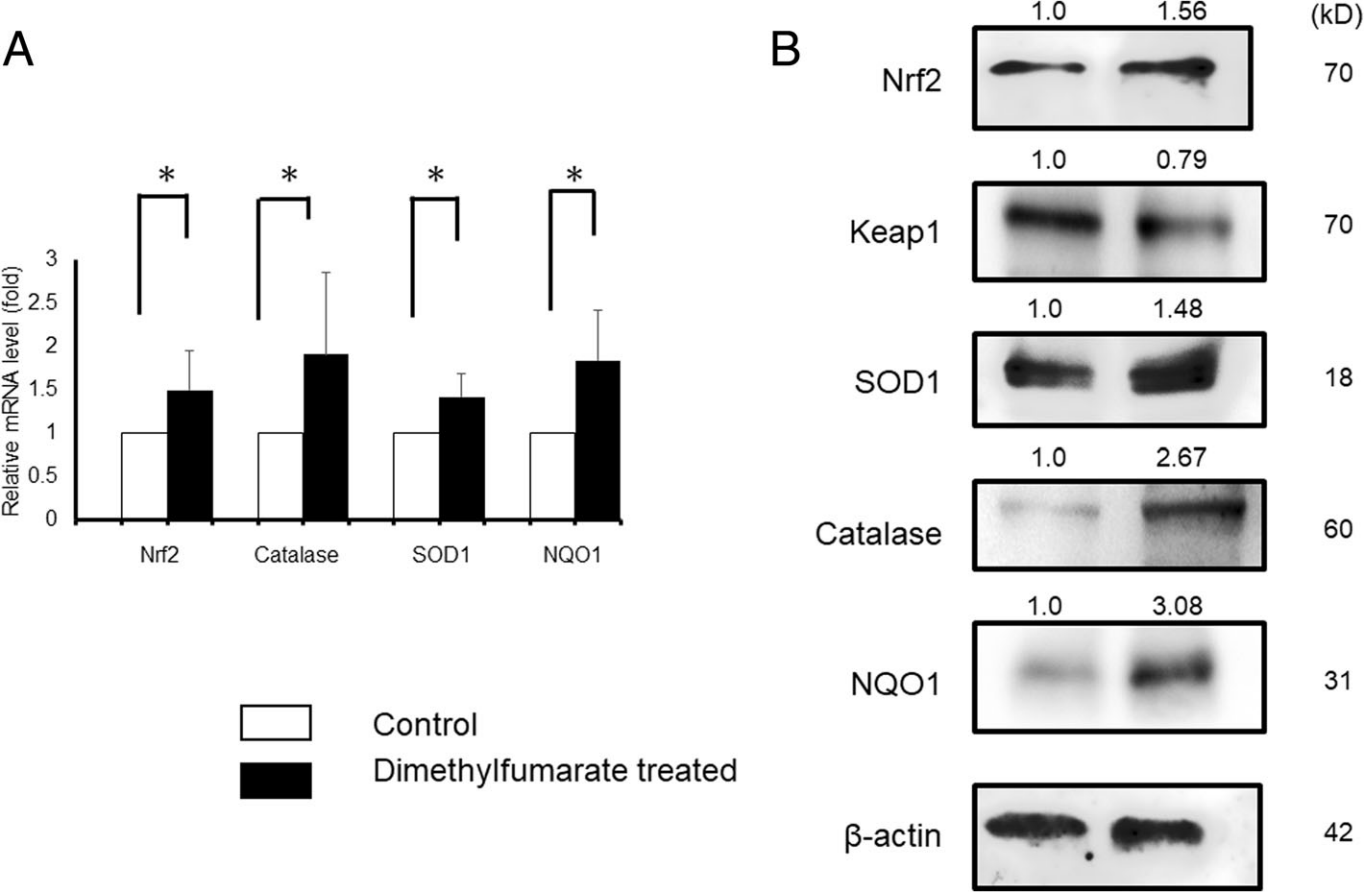
mRNA and protein levels of Nrf2 and antioxidants. Effect of DMF administration on the mRNA levels were investigated by qRT-PCR. The mRNA expression of Nrf2, catalase, SOD1, and NQO1 was normalized to RNA loading for each sample using GAPDH mRNA as an internal standard. DMF administration resulted in significant elevation of mRNA levels of Nrf2 (1.50-fold compared to control group, *p* < 0.05), catalase (1.91-fold compared to control group, *p* < 0.05), SOD1 (1.42-fold compared to control group, p < 0.05), and NQO1 (1.84-fold compared to control group, p < 0.05). The results are shown as the mean ± SD (bars) of 3 or 4 independent experiments. Simultaneously, DMF administration increased expression of Nrf2, catalase, SOD1, and NQO1 proteins in WB analysis. The protein levels of Keap1 was conversely decreased by DMF administration

DMF is known to activate the Nrf2/Keap1 pathway in various organs, resulting in elevation of antioxidants. To investigate the impact of DMF in the mouse ovaries, mice were administrated DMF, and mRNA and protein levels of Nrf2 and its target genes NQO1 [38–40], representative antioxidants catalase and SOD1 were investigated. We found that DMF administration to mice resulted in significant elevation of mRNA levels of Nrf2 (1.50-fold compared to control group), catalase (1.91-fold compared to control group), SOD1 (1.42-fold compared to control group), and NQO1 (1.84-fold compared to control group). Simultaneously, DMF administration increased expression of Nrf2 (1.56-fold compared to control group), catalase (2.67-fold compared to control group), SOD1 (1.48-fold compared to control group), and NQO1 (3.08-fold compared to control group) proteins in WB analysis. The protein levels of Keap1 (0.79-fold compared to control group) were conversely decreased by DMF administration (Fig. 5).

### DMF decreased OS in ovarian tissues

**Fig. 6:**
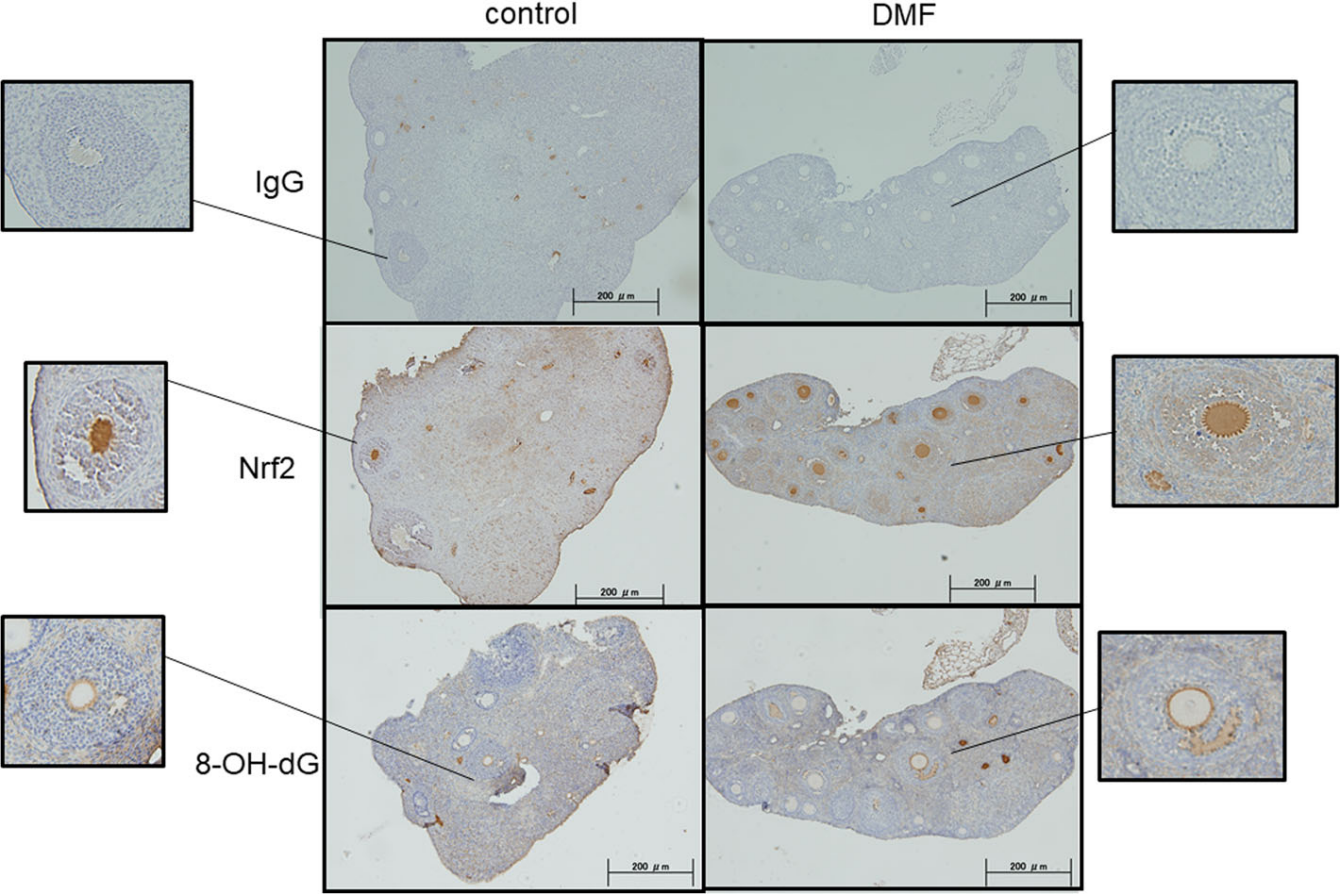
Expression of Nrf2 and 8-OHdG in mouse ovarian tissues. Immunohistochemical detection of Nrf2 and 8-OHdG proteins. Representative data from 6 specimens are shown. DMF administration increased expression in Nrf2, and conversely 8-OHdG expression was decreased

**Fig. 7:**
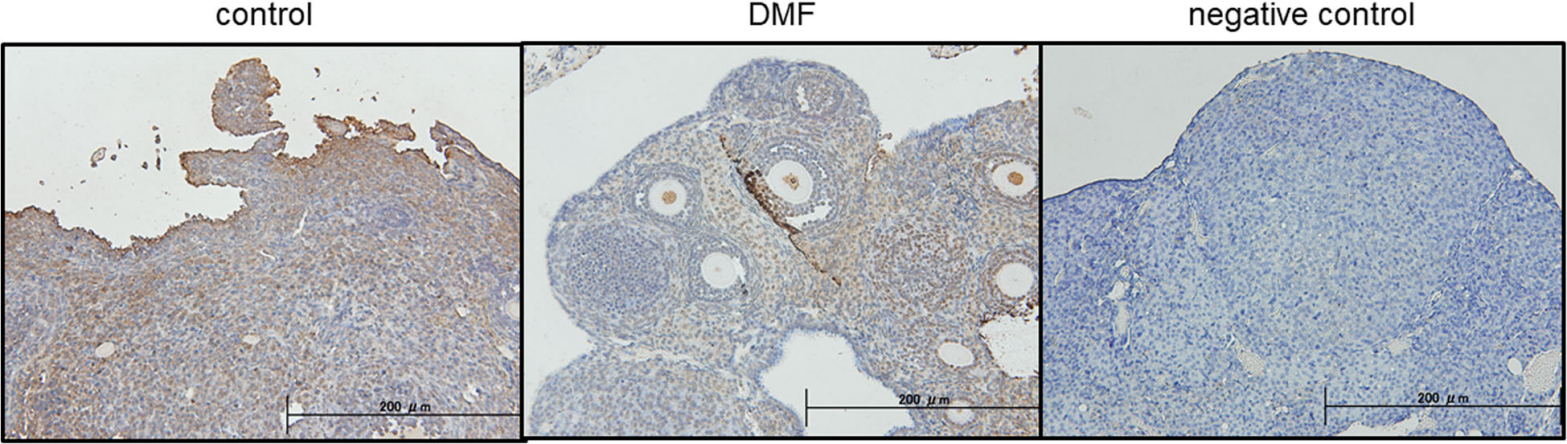
DNA damage in mouse ovarian tissues analyzed by TUNEL assay. Control group showed increased number of DNA damaged TUNEL-positive cells compared to DMF group. Representative data from 6 specimens are shown

To assess the impact of DMF administration, the expression of Nrf2 and 8-OHdG proteins in mouse ovarian tissues were confirmed by Immunohistochemistry (IHC). As shown in Fig. 6, DMF group showed increased expression in Nrf2, and conversely 8-OHdG which serves as an OS marker, showed decreased expression. DNA damage in the ovary was analyzed by TUNEL assay, and as shown in Fig. 7, control group showed increased number of expression of TUNEL-positive cells compared to DMF group.

### mRNA and protein levels of TERT and telomere were elevated by DMF administration

**Fig. 8:**
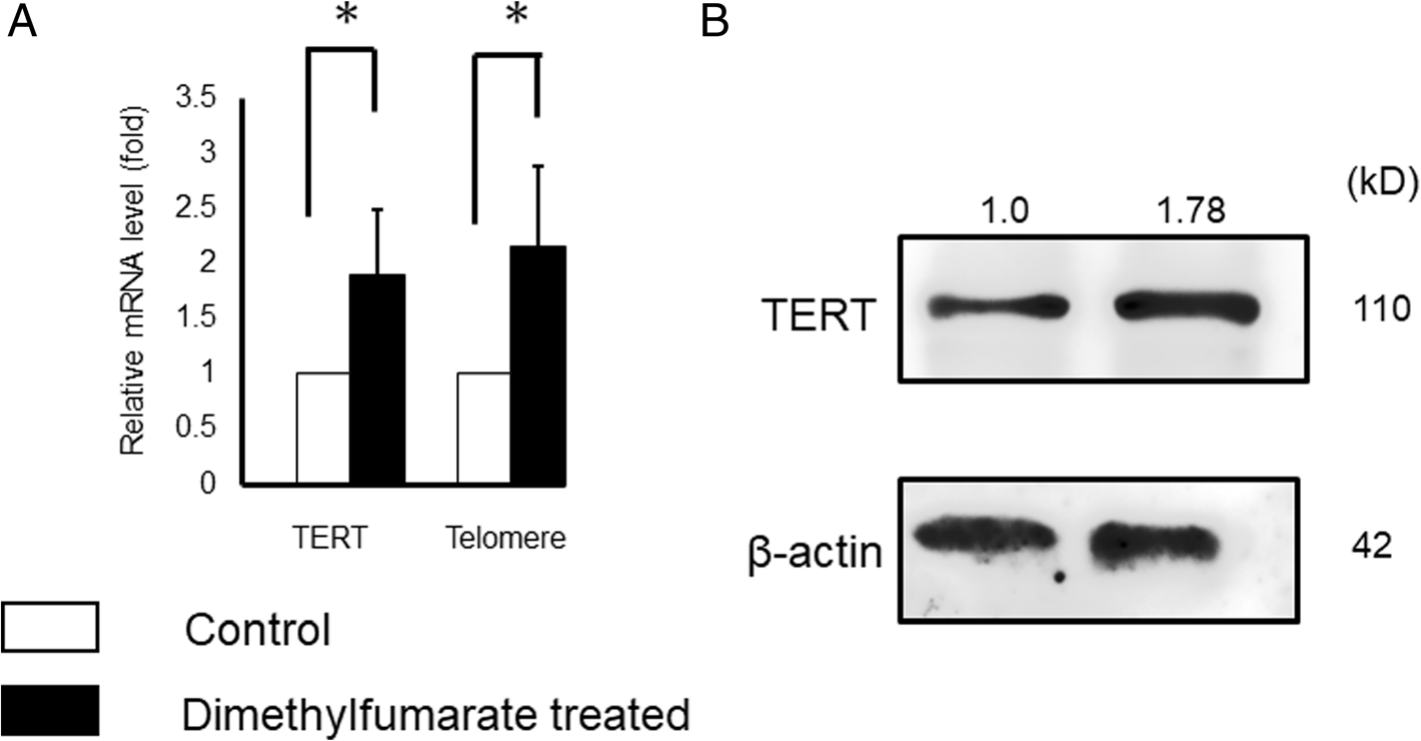
mRNA and protein levels of TERT and Telomere. Effect of DMF administration on the mRNA levels were investigated by qRT-PCR. The mRNA expression of Telomere and TERT was normalized to RNA loading for each sample using GAPDH mRNA as an internal standard. DMF administration resulted in significant elevation of mRNA levels of TERT (1.90-fold compared to control group, *p* < 0.05) and Telomere (2.15-fold compared to control group, p < 0.05). The results are shown as the mean ± SD (bars) of 3 or 4 independent experiments Simultaneously, DMF administration increased expression of TERT protein levels in WB analysis.

We found that DMF administration to mice resulted in significant elevation of mRNA levels of TERT (1.90-fold compared to control group) and Telomere (2.15-fold compared to control group), and TERT (1.78-fold compared to control group) protein levels were elevated in WB analysis (Fig. 8).

## Discussion

Nrf2/Keap1 pathway is a powerful defense mechanism against OS, and DMF is known to activate this pathway. DMF is a safe oral drug now used for treatment of relapsing MS in human, and no major adverse effects are reported [25]. Available literatures considered various dose of oral DMF administration in animal experiments [24, 32–34]. The maximum dose of Tecfidera® capsules used for MS patients is 480 mg/day, approximately 10 mg/kg/day, and animal studies (https://tec.ms-supportnavi.com/content/dam/commercial-jp/neurology/mssupportnavi/tecfidera/pdf/product/tecfidera_interview.pdf) confirmed that oral DMF administration dose of 50 and 100 mg/kg/day show no adverse reactions, while 250 mg/kg/day DMF administration resulted in weight loss of mice. Our previous study included various dosage of DMF [22], and from these results the dose 50 mg/kg/day was carefully selected in this study, and no adverse side effects including weight loss were observed.

Mouse ovary tissue sections were examined by IHC and TUNEL assay. As shown in Fig. 6, Nrf2 administration reduced 8-OHdG expression. 8-OHdG is one of the most commonly formed DNA lesions produced in response to OS, and is considered as a cellular marker for both OS and oxidative DNA damage [41], indicating decrease of OS by DMF administration. TUNEL assay (Fig. 7) shows that DMF administration results in decreased apoptosis cells.

The size of the remaining primordial follicle is the hallmark of mammalian female reproductive competence [42], and number of primordial follicles are important to estimate the length of the ovarian lifespan of an individual [43]. Our results in Fig. 3 show that with DMF administration, 60% more primordial follicles were preserved compared to control group, where no significant difference was seen in number of primary, secondary, and antral follicles. The size of the ovarian reserve varies between species, but the patterns of rise and fall in mouse and human ovary are similar [44]. Recently we have started to observe ovarian functions in young female taking DMF for MS treatment. Since precise follicle count is difficult in human, serum AMH levels which are known to reflect the size of the primordial follicle pool [45] are frequently used as prediction of ovarian reserve functions. Serum AMH levels as well as Nrf2 levels were significantly elevated (Fig. 4) in DMF administrated mice, and we will observe serum AMH and Nrf2 levels in female taking DMF as a prediction of ovarian reserve, along with antral follicle count.

Telomeres are structures that cap the ends of chromosomes and consist of tandem repeats of TTAGGG sequence that are associated with an array of proteins [46]. They protect chromosomes from degradation, and shorten with cell division and exposure to ROS [47]. Telomere length decreases with age, and number of studies report a relationship between reproductive aging and telomere lengths in human and mice [48–50]. Telomeres are especially sensitive to ROS [51], resulting in telomere shortening which leads to productive failure and infertility [52]. Telomere shortening can be reversed through the expression of an enzyme called telomerase, because telomerase is able to add de novo repeats onto chromosome ends [53]. Telomerase is a ribonucleoprotein that consists of TERT and the telomerase RNA component (TERC), and telomerase activity is mainly regulated with TERT activity [54]. Though the relation between telomere lengths and Nrf2 remains unclear, a study showed that in telomere-deficient mice, mitochondrial changes associated aging seem to be driven with suppression of Nrf2 [55]. Results in Fig. 8 show that mRNA and protein expressions of the TERT gene as well as mRNA expressions of telomerase were significantly elevated by DMF administration, and Nrf2 activation through DMF may be associated. However, since the telomere lengths have not been assessed yet, further investigation is required.

Presumably lifestyles of woman to delay childbearing in developed countries will persist, and more couples will undergo IVF treatment in the future. Literatures show that infertility is estimated to affect as many as 186 million people worldwide [4], and surprisingly couples in developing countries suffer as well as developed countries. However, IVF treatment is mostly inaccessible in developing countries, and patients are abandoned to their childless destinies. From these facts, a simple and safe treatment to preserve female ovarian reserve will be a marvel. Our results show that management of OS through activation of Nrf2/Keap1 pathway by oral DMF administration may lead to improved ovarian reserve, and this may become a solution for infertility treatment. Oral DMF is used in over 200,000 MS patients [25], and following the ovarian functions of female patients may lead to better understanding, and further research is required.

## Conclusions

In summary, we conclude that DMF administration activates the Nrf2/Keap1 pathway in the mouse ovary, elevate levels of antioxidants, and decrease DNA damage and OS, resulting in improved ovarian reserve in the mouse ovary. Studies of female patients receiving DMF could lead to management of OS in human ovaries, and further investigation is required.

## Abbreviations

8-OHdG: Human 8-hydroxy-2′-desoxyguanosine
AMH: Anti-mullerian hormone
ARE: Antioxidant response element
COC: Cumulus-oocyte complexes
COS: Controlled ovarian stimulation
DAB: 3,3-diaminobenzidine
DMF: Dimethylfumarate
ELISA: Enzyme-linked immunosorbent assay
GAPDH: Glyceraldehyde-3-phosphate dehydrogenase
H&E: Hematoxylin and eosin
hCG: Human chorionic gonadotropin
HTF: Human tubal fluid
IgG: Immunoglobulin G
IHC: Immunohistochemistry
IP: Intraperitoneal
IVF: In Vitro Fertilization
Keap1: Kelch-like ECH-associated protein 1
MS: Multiple sclerosis
NQO1: NAD(P)H quinone dehydrogenase 1
Nrf2: Nuclear factor-E2-related factor 2
OS: Oxidative stress
PBS: Phosphate buffered saline
POD: Peroxidase
qPCR: Quantitative polymerase chain reaction
ROS: Reactive oxygen species
SOD: Superoxide dismutase
TERC: Telomerase RNA component
TERT: Telomerase reverse transcriptase
TUNEL: Terminal deoxynucleotidyl transferase (TdT) dUTP Nick-End Labeling
WB: Western Blotting
